# Calcium Channel Blockers in Critical Care Medicine: Current Clinical Applications and Future Investigational Perspectives

**DOI:** 10.3390/medsci14020213

**Published:** 2026-04-25

**Authors:** Akram M. Eraky, Yasser Mokhtar, Guy Grabau, Adnan Khan, Ashish Acharya, Nichole Gadd, Mark Jarosz, Abhishek Premkumar

**Affiliations:** 1 Emergency Medicine Department, Freeman Health System, Joplin, MO 64804, USA; ngadd08@gmail.com; 2 Graduate Medical Education, Kansas City University, Kansas City, MO 64106, USA; 3 Pulmonology and Critical Care Medicine Department, Freeman Health System, Joplin, MO 64804, USA; ymmokhtar@freemanhealth.com (Y.M.); grabaumd@gmail.com (G.G.); dradnan84@gmail.com (A.K.); 4 Pulmonology and Critical Care Medicine Department, SUNY Upstate Medical University, Syracuse, NY 13210, USA; ashish.ach93@gmail.com; 5 Internal Medicine Department, Freeman Health System, Joplin, MO 64804, USA; mjarosz2163@gmail.com (M.J.); ab.shek2008@gmail.com (A.P.)

**Keywords:** calcium channel blockers, atrial fibrillation, hypertensive emergencies, pulmonary hypertension, vasospasm, hypertensive emergency, CHF

## Abstract

Calcium channel blockers (CCBs) are frequently used in the emergency department and intensive care unit for a wide range of critical conditions, including atrial fibrillation, hypertensive emergencies, acute pulmonary edema with sympathetic crashing, pulmonary hypertension, and vasospastic syndromes. However, their toxicity can lead to significant hemodynamic compromise, underscoring the importance of understanding their pharmacologic effects and safety profile. This review summarizes the current applications of CCBs in critically ill patients, evaluates their safety in congestive heart failure, and highlights emerging therapeutic roles and recent advances in the management of CCB toxicity.

## 1. Introduction

Calcium channel blockers (CCBs) block the L-type voltage-gated calcium channels in the myocardium, vascular smooth muscles, and pancreas; subsequently, blocking the inward calcium movement. CCBs are classified into non-dihydropyridine CCBs that are cardio-selective and affect mainly the myocardium and dihydropyridine CCBs that affect mainly the vascular smooth muscles. CCBs are lipophilic, highly protein-bound, and hepatically metabolized [1,2,3].

Non-dihydropyridines have negative inotropic and chronotropic effects, and they include diltiazem, which has minimal vasodilating effect, and verapamil, which has a potent vasodilating effect. Non-dihydropyridines are used mainly in the treatment of tachyarrhythmia. In contrast, dihydropyridine CCBs have their effect mainly on the vascular smooth muscles and minimally on the myocardium. As a result, they are used mainly in the treatment of hypertension, coronary vasospasm, cerebral vasospasm, high-altitude pulmonary edema, and migraines [1,4,5]. Table 1 summarizes the differences between non-dihydropyridine CCBs and dihydropyridine CCBs [1,2,3,4,5].

## 2. Discussion

CCBs are frequently used in the emergency department (ED) and the intensive care unit (ICU) to treat critically ill patients with various pathologies, such as atrial fibrillation (AF), hypertensive emergency, sympathetic crashing acute pulmonary edema (SCAPE), pulmonary hypertension, coronary vasospasm, and cerebral vasospasm. Additionally, CCBs’ toxicity may cause severe deterioration of hemodynamics. As a result, it is crucial to be familiar with CCBs’ usage in critically ill patients and their potential harm [4,6].

Herein, we also review the safety of the use of different types of CCBs in patients with congestive heart failure (CHF). In addition to that, we review the potential prospects of the use of CCBs in critically ill patients. This is, to our knowledge, the first review to discuss current uses of CCBs in the ICU, promising future uses, recent advances in managing CCB toxicity, and the safety of CCBs in patients with CHF.

A comprehensive narrative literature review was conducted to evaluate the current applications, safety, and emerging roles of calcium channel blockers (CCBs) in critically ill patients. A systematic search of electronic databases, including PubMed and Scopus, was performed.

The search strategy incorporated combinations of Medical Subject Headings (MeSH) terms and keywords, including but not limited to: “calcium channel blockers,” “dihydropyridine,” “non-dihydropyridine,” “critical care,” “intensive care unit,” “atrial fibrillation,” “hypertensive emergency,” “sympathetic crashing acute pulmonary edema (SCAPE),” “coronary vasospasm,” “cerebral vasospasm,” “pulmonary hypertension,” and “calcium channel blocker toxicity.” Boolean operators (“AND,” “OR”) were used to refine the search.

Studies published in English were considered, with priority given to original research articles, randomized controlled trials, observational studies, systematic reviews, meta-analyses, and relevant clinical guidelines. Reference lists of selected articles were also manually reviewed to identify additional pertinent studies.

Articles were selected based on their relevance to the clinical use of CCBs in critically ill populations, including their hemodynamic effects, therapeutic indications, safety in heart failure, and management of toxicity. Emphasis was placed on clinically applicable and contemporary evidence to ensure a focused and practical synthesis.

As this is a narrative review covering a broad spectrum of topics related to calcium channel blockers, rather than focusing on a narrowly defined question, a formal systematic review protocol and quantitative synthesis were not performed.

### 2.1. Dihydropyridine CCBs in Hypertensive Emergency

Hypertensive emergency is defined as having end-organ damage in the setting of systolic blood pressure of 180 or more and diastolic blood pressure of 120 or more, while hypertensive urgency has the same definition without having end-organ damage. The diagnosis of hypertensive emergency requires careful assessment and workup to rule out end-organ damage, as there is no specific blood pressure threshold that reliably predicts this condition [7].

Many antihypertensives could be used in the management of acute hypertensive emergencies. CCBs such as nicardipine and clevidipine are commonly used first-line agents in many hypertensive emergencies, particularly due to their titratability, favorable hemodynamic effects, and safety profile [8,9,10,11,12,13,14].

Nicardipine and clevidipine are the two dihydropyridine CCBs, which are available as continuous venous infusion in the ICU [14,15,16]. Many studies show that there is no significant effectiveness difference between nicardipine and clevidipine [17,18,19]. However, clevidipine could be effective in some patients who are resistant to nicardipine [20]. Table 2 demonstrates the differences between nicardipine and clevidipine [16,18,21,22,23,24].

It is important to notice that some antihypertensives are preferred over CCBs in some pathologies. In cocaine toxicity, benzodiazepines are preferred as a first-line therapy to offset the sympathetic drive triggered by cocaine. In aortic dissection, beta-blockers are preferred to control both heart rate and blood pressure and prevent reflex tachycardia that can be associated with other anti-hypertensives. Dihydropyridine CCBs can be used when the first-line treatment is contraindicated or cannot achieve the blood pressure target alone [25,26,27,28]. Some antihypertensives are contraindicated in some pathologies, and they may cause an increase in blood pressure. For example, beta-blockers are contraindicated in cocaine abuse and pheochromocytoma crisis, before starting an alpha antagonist, as they may cause an unopposed alpha agonist effect [28,29,30].

Blood pressure should be gradually reduced. Systolic pressure should be reduced by not more than 25% during the first hour and not less than 160/100 over the next two to six hours. There are two exceptions to this rule. The first exception is that blood pressure should be reduced aggressively to target a systolic blood pressure less than 120 within the first hour in aortic dissection and less than 140 in pheochromocytoma crisis and hemorrhagic stroke. The second exception is that in ischemic stroke, permissive hypertension is allowed up to 220/120 if the patient is not treated with thrombolytics, or up to 185/110 if the patient is a candidate for thrombolytics [7].

### 2.2. Dihydropyridine CCBs in Patients with CHF

There is a theoretical concern that CCBs should be avoided in patients with heart failure (HF) because of their negative inotropic effect. However, dihydropyridine CCBs have only a minimal ionotropic effect as they are vascular-selective [31]. Some limited studies showed that despite their negative inotropic effect, dihydropyridine CCBs may increase ejection fraction (EF) and cardiac index (CI) by decreasing the afterload [32,33]. Additionally, a recent study showed that Nicardipine is safe for the management of hypertensive emergency in patients with a history of reduced ejection fraction (EF) and presenting with acute heart failure (AHF) with hypertension [34]. More clinical studies are encouraged to investigate the effect of dihydropyridine CCBs on cardiac physiology in patients with a history of heart failure with reduced ejection fraction (HFrEF).

### 2.3. Dihydropyridine CCBs in Sympathetic Crashing Acute Pulmonary Edema (SCAPE)

In SCAPE, an acute increase in peripheral resistance and hypertension may limit the left ventricular pumping, leading to increased filling pressures, acute heart failure, and pulmonary edema. SCAPE is also known as AHF with hypertension. For patients with SCAPE, besides non-invasive positive pressure ventilation (NIPPV), IV nitroglycerin is considered the first-line treatment due to its effective venous vasodilation and subsequent reduction in preload and pulmonary edema, as well as its effect on afterload reduction in high doses [35,36]. According to the American Heart Association (AHA) guidelines, the use of nitroglycerin and other vasodilators is recommended in acute decompensated heart failure patients with mitral regurgitation, acute coronary syndrome, or hypertension [37].

Theoretically, the use of dihydropyridine CCBs in SCAPE is reasonable as a second line after failing nitroglycerin [38]. Some studies showed that dihydropyridine CCBs are safe in patients with SCAPE [34,39]. One observational study compared the use of Nicardipine versus nitroglycerin for SCAPE. Nicardipine was found to be more effective than nitroglycerin regarding frequency of adding additional anti-hypertensives and time to obtain optimal blood pressure [40]. More clinical trials and more observational studies are encouraged to investigate the effect of different vasodilators in the management of SCAPE in different populations.

### 2.4. Non-Dihydropyridine CCBs as a Rate Control Agent in Afib

In the outpatient long-term management of atrial fibrillation (Afib), rhythm control had no significant mortality benefit compared to rate control [41,42]. In patients with HFrEF and symptomatic patients, rhythm control should be attempted to rule out arrhythmia-induced cardiomyopathy as a cause of reduced EF or patients’ symptoms [43,44,45]. In contrast to outpatient management, the choice between rhythm control and rate control depends mainly on the clinical presentation and hemodynamic stability in the inpatient management of Afib [43,46].

In hemodynamically unstable patients with Afib with rapid ventricular response (RVR), acute rhythm control with electrical cardioversion is recommended. In hemodynamically stable patients, beta blockers or non-dihydropyridine CCBs are recommended for acute rate control [43,46,47,48]. IV magnesium can be added to the rate-controlling agents as an adjunctive agent, as it was found to be superior to rate-controlling agents alone due to its ability to block the slow inward calcium channels in the sinoatrial (SA) and atrioventricular (AV) nodes [43].

### 2.5. Non-Dihydropyridine CCBs in Patients with CHF

**Figure 1:**
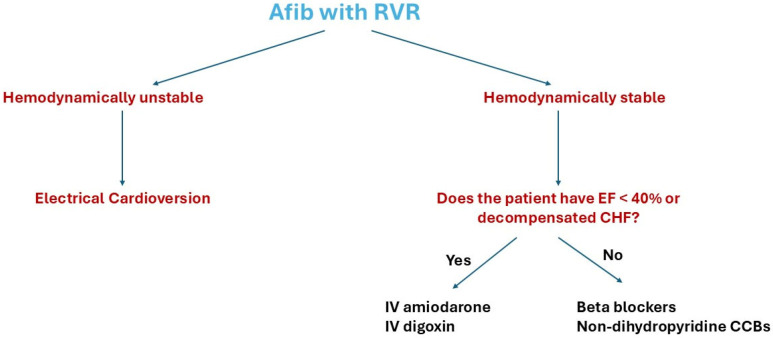
The management of Afib with RVR according to the AHA guidelines. CCBs, calcium channel blockers; Afib, atrial fibrillation; RVR, rapid ventricular rate; EF, ejection fraction; IV, intravenous.

In patients with decompensated HF, non-dihydropyridine CCBs should be avoided to control the rate in Afib with RVR. Also, non-dihydropyridine CCBs are contraindicated in patients with severely reduced ejection fraction (EF) (EF < 40%), regardless of the presence of decompensation, due to their negative inotropic effect [43,46]. Many studies suggested an association between the use of non-dihydropyridine CCBs in patients with HF and Afib with RVR and increased mortality [43,46,49,50,51]. When non-dihydropyridine CCBs and beta-blockers are contraindicated or ineffective, IV amiodarone or IV digoxin should be tried for acute rate control [43,46] (Figure 1).

### 2.6. Non-Dihydropyridine CCBs in Patients with SVT

CCBs, particularly the non-dihydropyridine agents like verapamil and diltiazem, are effective in the acute and chronic management of supraventricular tachycardias (SVTs) that depend on atrioventricular (AV) nodal conduction, such as AV nodal reentrant tachycardia (AVNRT) and AV reentrant tachycardia (AVRT). By inhibiting L-type calcium channels in the AV node, they slow conduction velocity and prolong the refractory period, thereby interrupting reentrant circuits and controlling the ventricular response [52,53,54].

Intravenous verapamil or diltiazem is commonly used for acute termination when vagal maneuvers or adenosine are ineffective or contraindicated. Oral formulations are useful for rate control and prevention of recurrence in patients who are not candidates for catheter ablation [52]. CCBs should be avoided in patients with pre-excited atrial fibrillation (e.g., Wolff–Parkinson–White pattern) due to the risk of facilitating rapid conduction through the accessory pathway and precipitating ventricular fibrillation [52,54].

### 2.7. CCBs in Pulmonary Hypertension

CCBs have been used in the long-term outpatient management of pulmonary hypertension in subtypes that are responsive to vasoreactivity testing. However, the studies discussing the use of IV CCBs in critically ill patients with pulmonary hypertension are scarce [55,56,57,58,59,60]. Only one case report was found discussing the use of CCBs in decompensated patients with vasoactive pulmonary arterial hypertension [61].

The effect of CCBs on cardiovascular hemodynamics in chronic pulmonary hypertension without stratification to specific subtypes is discouraging. Administration of nifedipine was found to increase right ventricular end-diastolic pressure (RVEDP) and decrease the right ventricular contractility without causing significant pulmonary vasodilatation in patients with chronic pulmonary hypertension [59,60]. This means CCBs may deteriorate the right heart function without decreasing the pulmonary arterial blood pressure. In acute cases, this may deteriorate the patients’ condition. In another study, the CCB-induced decrease in afterload compensated for the decreased contractility triggered by CCBs in chronic hypertension [62]. However, this makes CCBs nonideal for acute decompensated hypotensive patients.

Treatment of hypotensive patients with acute right heart failure due to acute pulmonary hypertension consists of: (1) reversal of the triggering causes, such as pulmonary embolism, (2) increasing preload with fluids, (3) maintaining systemic blood pressure to maintain organs’ perfusion with vasopressors, (4) increasing right ventricular contractility with inotropes, and (5) decreasing the pulmonary hypertension with vasodilator, such as inhaled nitric oxide, Milrinone, prostaglandins, and phosphodiesterase-5 inhibitors [60].

Using IV CCBs as a pulmonary vasodilator is appealing in patients who respond to vasoreactivity testing in some phenotypes of acute on chronic pulmonary hypertension in decompensated patients [61]. However, this pulmonary vasodilation effect is achieved only in some phenotypes, and giving it to patients who would not get the pulmonary vasodilation effect could worsen the patient’s right heart failure by decreasing right heart contractility [59,60].

A calcium-sensitizing agent, such as Levosimendan, could be a promising treatment in patients with pulmonary hypertension and acute decompensated right heart failure because of its effect on increasing the right ventricular contractility and the pulmonary vasodilation in patients with connective tissue disease-associated pulmonary arterial hypertension [63,64]. This indicates that calcium-sensitizing agents, such as Levosimendan, have a more promising role in the management of acute right heart failure than CCBs.

Given the scarcity of the use of both CCBs and calcium-sensitizing agents in critically ill patients with acute right heart failure and pulmonary hypertension, more clinical studies are encouraged to investigate their role in different phenotypes of pulmonary hypertension.

### 2.8. CCBs in the Treatment of Vasospasms

CCBs can be used in both cardiac and cerebral vasospasms. Nimodipine is a second-generation CBB that is highly lipid-soluble and can cross the blood–brain barrier (BBB) easily. Nimodipine is used in the prevention and treatment of cerebrovascular spasm for its vasodilatation ability and anti-free radical effect. Additionally, Nimodipine may have a potential neuroprotective effect in ischemic neurons by preventing calcium overload and neurotoxicity [65,66].

There is no significant difference found between oral and intravenous nimodipine in efficacy in the prevention of cerebral vasospasm following subarachnoid hemorrhage [67]. A recent clinical trial investigated the use of intraventricular nimodipine in patients with aneurysmal subarachnoid hemorrhage. There was no significant difference in efficacy between intraventricular and oral nimodipine [68].

CCBs are also known to be the cornerstone in the treatment of cardiac vasospasms, such as post-cardiac transplant coronary vasospasm, immunosuppressant-induced coronary vasospasm, and vasospastic angina [69,70,71]. Although contemporary management of cardiac allograft vasculopathy has shifted toward immunomodulatory and antiproliferative therapies, CCBs continue to play an adjunctive role by improving coronary vasomotor function, mitigating calcineurin inhibitor–induced vasoconstriction, and addressing the dynamic component of transplant vasculopathy [71,72,73,74]. More clinical studies are encouraged to investigate the role of CCBs in cardiac allograft vasculopathy.

### 2.9. CCBs in the Treatment of High-Altitude Pulmonary Edema (HAPE)

High-altitude pulmonary edema (HAPE) usually occurs at heights of more than 3000 m. HAPE develops as noncardiogenic edema that results from hypoxic pulmonary vasoconstriction that varies in the pulmonary vasculature and subsequent pulmonary hypertension. The main treatment of HAPE includes descent and oxygen supplementation. CCBs, such as nifedipine, have been shown to reduce pulmonary hypertension and improve oxygenation and could be used as an adjunctive treatment in HAPE management, besides the first-line treatment, including descent and oxygen supplementation [75,76,77,78].

The recent guidelines by the Wilderness Medical Society suggest the use of nifedipine in the treatment of HAPE if descent and oxygen are not available. If nifedipine is not available, they recommend using tadalafil or sildenafil. They also recommend the use of nifedipine for HAPE prevention in people who are susceptible to HAPE [75].

### 2.10. CCB Toxicity

CCB overdose leads to suppression of the sinoatrial node (SA) and the atrioventricular node (AV), resulting in bradycardia and subsequent hypotension. It may cause vasodilation too, leading to hypotension. The effect of CCBs can vary based on the type of CCBs. Dihydropyridine CCBs mainly cause vasodilation with less effect on the myocardium, while non-dihydropyridine CCBs mainly cause decreased myocardial contractility and heart rate. Verapamil is a non-dihydropyridine CCB that has both a potent vasodilating effect and a negative inotropic and chronotropic effect [79,80,81].

First-line treatment of CCB toxicity includes calcium salts, IV fluids, high-dose insulin, and vasopressors. Atropine is also suggested as a first-line treatment in patients with CCB toxicity and unstable bradycardia. Regarding hemodialysis, most of the CCBs have hepatic metabolism and are lipophilic. As a result, hemodialysis and hemofiltration are not effective in the management of CCB toxicity [79,82]. If the patient is refractory to the first-line treatment, a pacemaker is recommended for unstable bradycardia, IV lipid-emulsion therapy, and Veno-arterial Extracorporeal Membrane Oxygenation (VA-ECMO) in refractory cases [82,83].

Vasopressor administration should be guided by the type of shock. Both norepinephrine and epinephrine are recommended to increase contractility and offset the vasodilatation. Dobutamine is recommended in the presence of myocardial dysfunction. Patients with CCBs may also have mixed shock and require more than one pressor. Dopamine is not recommended due to its inconsistent hemodynamic effects in other studies. Pressors that have a selective vascular effect, such as vasopressin and phenylephrine, and do not have ionotropic and chronotropic effects should be avoided as a first-line pressor, because they may worsen the cardiogenic shock by increasing the afterload without increasing the contractility [82,83,84].

There is no clear consensus regarding whether to start vasopressors or high-dose insulin first [85,86]. In an animal RCT, starting vasopressors and high-dose insulin at the same time had a more potent effect compared to high-dose insulin alone in beta-blocker toxicity [87]. It is also important to avoid pressors with beta-agonist effect in severe aortic stenosis and hypertrophic cardiomyopathy, as they may decrease the left ventricular volume, stroke volume, and cardiac output, leading to exacerbating the patient’s shock. Those patients may benefit from high-dose insulin and phenylephrine [88]. More clinical studies are encouraged to compare whether starting pressors or high-dose insulin first is more effective in patients with toxicity of CCBs.

A potential treatment for CCB toxicity is calcium-sensitizing agents, such as Levosimendan, which could be a promising option due to their effect on increasing cardiac contractility. However, it should be used with other pressors to offset its vasodilatory effect, which may worsen hypotension [89,90]. In a recent experimental study, the combination of levosimendan and calcium chloride was found to increase the survival rate in CCB toxicity [91]. Clinical studies are encouraged to explore this era and examine the effect of using Levosimendan on hemodynamics in patients with CCB toxicity.

## 3. Conclusions

CCBs are frequently used in the ED and ICU to control heart rate, SCAPE, or hypertension in critically ill patients. It has other uses beyond its regular uses, such as the off-label use in HPAE, cerebral vasospasm, migraines, and pulmonary hypertension. It is essential to be familiar with CCBs’ common uses in the ED and ICU and their potential harm in specific groups, such as patients with CHF. Additionally, CCBs are also considered a potentially promising treatment in many conditions, such as pulmonary hypertension, and more clinical studies should be encouraged to investigate the potential benefits and risks of CCBs in critically ill patients.

## Figures and Tables

**Table 1:** Differences between non-dihydropyridine CCBs and dihydropyridine CCBs.

Feature	Dihydropyridines	Non-Dihydropyridines
Primary Site of Action	Vascular smooth muscle	Myocardium and SA/AV nodes
Mechanism of Effect	Potent vasodilation	Negative inotropy and chronotropy
Effect on Heart Rate	Reflex tachycardia possible	Decrease heart rate
Effect on Contractility	Minimal	Decreased (negative inotropic effect)
Effect on AV Node	Minimal	Significant slowing (AV nodal blockade)
Hemodynamic Effect	↓ Systemic vascular resistance	↓ HR, ↓ contractility, mild vasodilation
Common Clinical Uses	Hypertension, cerebral vasospasm, HAPE, migraines	Atrial fibrillation/flutter, SVT, rate control
Examples	Amlodipine, Nifedipine, Nicardipine, Clevidipine	Diltiazem, Verapamil
Use in Heart Failure (HFrEF)	Generally safer (caution still needed)	Typically avoided (due to negative inotropy)
Toxicity Profile	Profound vasodilation → hypotension, reflex tachycardia	Bradycardia, heart block, cardiogenic shock

Abbreviations: CCB, calcium channel blocker; SA, sinoatrial; AV, atrioventricular; HR, heart rate; HFrEF, heart failure with reduced ejection fraction; SCAPE, sympathetic crashing acute pulmonary edema; SVT, supraventricular tachycardia; HAPE, high altitude pulmonary edema; ↓, decrease.

**Table 2:** Differences between nicardipine and clevidipine as antihypertensives used in the ICU as intravenous infusion drips.

Feature	Nicardipine	Clevidipine
Drug class	Second-generation, dihydropyridine CCB	Ultra-short-acting, third-generation, dihydropyridine CCB
Mechanism	Arterial vasodilation → ↓ systemic vascular resistance	Selective arterial vasodilation → ↓ systemic vascular resistance
Onset of action after starting infusion	5–10 min	2–4 min
Offset of action after stopping infusion	30–60 min	5–10 min
Half-life	30–60 min	~1 min
Metabolism	Hepatic metabolism (CYP3A4)	Rapid hydrolysis by blood and tissue esterases
Titration speed	Moderate	Very rapid titration possible
Formulation	Aqueous IV solution	Lipid emulsion (similar to propofol)
Infusion dosing	Start 5 mg/h, increase by 2.5 mg/h every 5–15 min (max ~15 mg/h)	Start 1–2 mg/h, double every 90 s until near goal, then titrate (max ~32 mg/h)
BP control speed	Fast	Faster than nicardipine in some studies
Effect on cardiac contractility	Minimal negative inotropy	Minimal negative inotropy
Special advantages	Widely available, inexpensive	Ultra-short acting → precise BP control
Key disadvantages	Longer offset time	Expensive
Important contraindications	Advanced aortic stenosis	Advanced aortic stenosis and Egg or soy allergy (lipid formulation)

Abbreviations: CCB, calcium channel blocker; BP, blood pressure; ↓, decrease.

## Data Availability

No new data were created or analyzed in this study.
